# Blockchain-based privacy and security model for transactional data in large private networks

**DOI:** 10.1038/s41598-023-44101-x

**Published:** 2023-10-10

**Authors:** Bello Musa Yakubu, Jamilu Sabi’u, Pattarasinee Bhattarakosol

**Affiliations:** 1 Department of Mathematics and Computer Science, Faculty of Science, Chulalongkorn University, Bangkok, Thailand; 2 Department of Mathematics, Yusuf Maitama Sule University, Kano, Kano State Nigeria

**Keywords:** Engineering, Mathematics and computing

## Abstract

Cyberphysical systems connect physical devices and large private network environments in modern communication systems. A fundamental worry in the establishment of large private networks is mitigating the danger of transactional data privacy breaches caused by adversaries using a variety of exploitation techniques. This study presents a privacy-preserving architecture for ensuring the privacy and security of transaction data in large private networks. The proposed model employs digital certificates, RSA-based public key infrastructure, and the blockchain to address user transactional data privacy concerns. The model also guarantees that data in transit remains secure and unaltered and that its provenance remains authentic and secure during node-to-node interactions within a large private network. The proposed model has increased the encryption speed by about 17 times, while the decryption process is expedited by 4 times. Therefore, the average overall acceleration obtained was 16.5. Both the findings of the security analysis and the performance analysis demonstrate that the proposed model can safeguard transactional data during communications on large private networks more effectively and securely than the existing solutions.

## Introduction

Nodes connected to the Internet of Things (IoT) may gather user data from their environs, distribute that data among themselves, and communicate with other embedded software systems in their networks^1^. They create a substantial amount of user data, and nodes may not always be able to rely on one another during crucial communications. The broad transmission of private information and the exposure of user routines and preferences via the usage of internet-connected nodes can raise serious privacy problems^2^. Since adversaries may conduct active or passive attacks, such as man-in-the-middle (MITM) and replay attacks, on the network, when such data is disseminated across a large private network such as a smart marketplace, smart grid network, vehicular ad hoc network, etc., the privacy of users is especially at stake^3,4^.

Creating a secure environment for each component of the IoT architecture is unquestionably one of the most challenging aspects of IoT private networks^5^. However, many solutions have been proposed to address the present issues with large private networks. Prior to securing a private IoT network, it is necessary to evaluate the security of each IoT component. Such IoT private networks consist mostly of connected nodes, gateways that enable node connections, network infrastructures, and cloud infrastructures^5,6^. The sheer number of networked nodes and the diversity of resources they represent make the IoT an enticing target for adversaries; also, centralized security solutions cannot keep up with the amount of data being processed and stored.

Each component of the IoT architecture has the potential to become a bottleneck or failure point, which might disrupt the whole network^5,7^. Among the damaging attacks that can be performed against IoT nodes private networks are hacking, data theft, and remote hijacking. Unauthorized users can get access to the system and steal, modify, or delete data. If an adversary gains access to an IoT node that is linked to a server, all other nodes connected to that server become exposed if the server is compromised. To sum up, current IoT systems are susceptible to issues related to data integrity and privacy, manipulation, node impersonation, and unauthorized data access during interactions, among others^8,9^. In this context, blockchain technology, in conjunction with digital certificates and integer factorization-based public key infrastructure (PKI) such as the RSA (Rivest–Shamir–Adleman) algorithm, could facilitate the development of IoT communications that protect user data privacy and mitigate other identified security concerns^10,11^.

Scalability and single point of failure Timestamping, anonymity, trust, and dependability are a few of the IoT security concerns that the use of digital certificates and PKI in an Ethereum blockchain network could help resolve^8–10,12^. In addition, it can offer IoT nodes a simple framework for transmitting data in a reliable, consistent, and contractually guaranteed way. Message exchanges between IoT nodes can be enabled via the use of smart contracts, which represent the communicating nodes agreements. These qualities facilitate the autonomy of the nodes and the development of artificial intelligence systems in the private network.

This study proposes a security framework that uses digital certificates, RSA-based PKI, and the Ethereum blockchain to address user transactional data privacy concerns and to ensure that data in transit remains secure and unaltered and that its provenance remains authentic and secure during node-to-node interactions within a large private network. The following are the significant contributions of this study:

I. A blockchain-based privacy and security model for transactional data on large private networks is presented. The model accomplishes its privacy and security objectives via the use of three essential processes: message packaging and padding, message encryption and signing, and signature verification and decryption. This makes the approach very adaptable and secure for large private networks.
II. The model employs digital certificates, RSA-based public key infrastructure, and the Ethereum blockchain. In addition to addressing user transactional data privacy concerns and ensuring that data in transit stays safe and unmodified, the system also guarantees that data provenance remains legitimate and secure throughout node-to-node interactions within a large private network.
III. To examine the security, viability, and efficacy of the proposed model, theoretical evaluations of system performance and security were undertaken in this study. Using the model, a prospective message sender can successfully deliver its message package to the intended network recipient in a secure and private manner with low computational expense, according to the evaluation findings.

The rest of the paper is structured as follows: the related works are described in Section “Related works”. The system model is provided in Section “System model”. The proposed model was discussed in Section “The proposed model”. The security evaluation is covered in Section “Security analysis”. Section “Performance analysis” describes performance evaluation. Section “Discussions and future improvements” provides discussions and future improvement, while section “Conclusion” concludes the paper.

## Related works

Transactional data privacy preservation is the practice of preventing unauthorized users from disclosing personal data while processing it via networks^13^. There are five kinds of privacy-preserving approaches: encryption-based^14,15^, perturbation-based^16,17^, authentication-based^18,19^, differential privacy^20,21^, and blockchain-based^22,23^. Each of them is addressed individually.

Based on encryption, several privacy-preserving techniques, such as Refs.^14,15,24–26^, have been developed to allow the encryption of data during message exchange. Most schemes rely on symmetric, asymmetric, or homomorphic encryption techniques^27^. For instance, in Ref.^24^, a location-based symmetric key generator was utilized to protect the location of service providers during peer-to-peer interactions. The technique is utilized to coordinate a session key for the selection of a target range service provider. Due to the dearth of session key privacy protection, however, it becomes a vulnerable target and is susceptible to attacks and leakage. Similarly, symmetric searchable encryption (SSE), another session key technique, was used in Ref.^25^ to encrypt both the public and private portions of electronic medical records separately in order to accomplish access control and data privacy during patient data sharing. Attribute-based encryption technology was employed to address the session key privacy protection issues. Due to the double encryption employed in this instance, the system is prone to high computational complexity. Technique^26^ demonstrates the use of a smart contract token-based solution and a lightweight post-quantum encryption algorithm known as Nth-degree Truncated polynomial Ring units (NTRU) to address issues related to users' data security and privacy concerns. This technique was used to accomplish access control and user data privacy during interactions. Despite advancements in these encryption methods that provide mathematical computations on encrypted data, fewer application areas adopt these methods owing to their high computing requirements and restricted operating capabilities^28^.

Numerous methods have also utilized privacy-preserving strategies based on perturbation^16,17^. They primarily use data transformation techniques, like statistical and data forecast measurements, to disguise sensitive data in new forms^29^. The most difficult aspect of these techniques is striking a balance between data value and privacy protection. Ideally, both are necessary; however, these requirements are inverse, hence complete privacy protection and optimal data usefulness cannot coexist^30^.

Several further methods, such as Refs.^18,19^, have embraced authentication-based privacy-preserving methods. They are mostly used to provide authentication procedures for users and systems, such as single sign-on, federated identity, and key management^31^. These methods are not relevant to cyberphysical system protocols, though. Similarly, a Chebyshev Chaotic-Map-based single-user sign-in (S-USI) system was used in Ref.^32^. The system employs S-USI to secure a sensor-based or sensor-tag-based intelligent healthcare environment. Authentication is strengthened by the presentation of a secure S-USI approach and coexistence protocol evidence for ubiquitous cloud services. Since they are only intended for authentication, such authentication-based privacy preservation systems cannot be utilized to safeguard data sent over huge private networks^33^.

Numerous methods, including^20,21^, also used differential privacy measures. Using effective statistical approaches, such as Gaussian and Laplace processes, to thwart inference and data poisoning threats is their primary objective. Differential privacy techniques provide perfect privacy since they make no assumptions about the knowledge of the attacker^34^. The techniques also guarantee that disconcerted computations of data will not significantly change when the actual data are modified^33,34^. Differential privacy results may exacerbate vulnerabilities, and not all algorithms are compatible with the notion of wide-open, large private networks. Likewise, differential privacy provides only statistical guarantees that the difference between real and fuzzy data is limited to epsilon. Consequently, differential privacy queries may disclose a small amount of information whose loss might be catastrophic if an attacker can repeatedly make similar requests^35^.

Recent blockchain-based approaches that protect privacy include^4,22,23^. Blockchain, a peer-to-peer crypto link, can be used to safeguard data transfers or network nodes^10^. Peers from distant networks serve as nodes and can help in solving a hash-based puzzle challenge to assure transaction integrity. Transaction records were compacted to form a block of transactions, and a ledger contains all the generated blocks. Since all blocks are updated simultaneously, every peer has a copy of the same ledger^36,37^. Proof of work (PoW) and proof of stake (PoS) are used by Bitcoin and Ethereum, respectively, to verify transactions and produce new blocks^10^. PoW depends on processing power to solve the puzzle challenge, whereas PoS employs a deterministic method that sometimes loses blocks^38^. When an adversary miner has at least 51% more processing power than other network nodes, it can execute a 51% attack against both approaches^38,39^.

Given the novel proof of authority (PoA) consensus algorithm introduced by Ethereum to handle 51% attack vulnerabilities, several alternatives were proposed to combine blockchain technology with one or more of the previously mentioned privacy-preserving strategies to address data privacy issues on large private networks^10,38,39^. For example, the authors in Ref.^40^ present a blockchain-based solution for smart grid privacy breaches, while^41^ provided blockchain-enabled deferential privacy-based network solutions for data privacy regulations. Likely, the authors in Ref.^42^ created a support vector machine method to identify invasive actions in large private networks and used blockchain to validate data sources. Furthermore, authors in Ref.^43^ have developed a distributed blockchain-based method to safeguard private networks against cyber intrusions that result in data privacy concerns. However, owing to the range of privacy approaches, combining these solutions into a blockchain-edge computing platform without addressing the blockchain's transparency aspects would pose fundamental security difficulties^33^. Ernest and Shiguang^4^ attempted to achieve privacy without compromising blockchain transparency. Using randomly generated public keys and digital signatures, the authors offer a privacy-aware approach based on the elliptic curve cryptosystem (ECC) that protects user privacy in blockchain-edge computing. Their research was promising, but due to computational needs, it cannot be instantly deployed to heterogeneous nodes in smart private networks. Therefore, given the major advantages of blockchain, there is a need to develop a better solution that can combine these aspects with other cryptographic approaches to handle the pending challenges of transactional data privacy preservation more effectively. An overview of the most notable and currently relevant studies is provided in Table 1.

**Table 1:** Overview of the related works.

Techniques	Objectives	Limitations
Encryption^14,15,24–27^	To enable encryption of data during message exchange	High computation overhead and restricted operating capabilities
Perturbation^17,18^	To conceal sensitive data in new forms	Striking a balance between privacy protection and data value
Authentication^18,19,32^	To provide user or data privacy protection via authentication procedures during interactions	Cannot guarantee data privacy sent over huge private networks
Differential privacy^20,21^	To provide perfect data privacy and guarantee less significant data modification	A small amount of data is disclosed, which can be readily significant over time
Blockchain^4,22,23^	To safeguard network nodes or data transfers	Vulnerable to a 51% attack and double spending
Blockchain, differential privacy, machine learning, and encryption^40–43^	To address data privacy issues on large private networks	Bottleneck due to the blockchain transparency feature
Blockchain, PKI, elliptic curve cryptosystem (ECC)^4^	To achieve privacy without compromising blockchain transparency	High computation overhead and cannot be readily deployed to heterogeneous nodes in smart private networks

## System model

**Figure 1:**
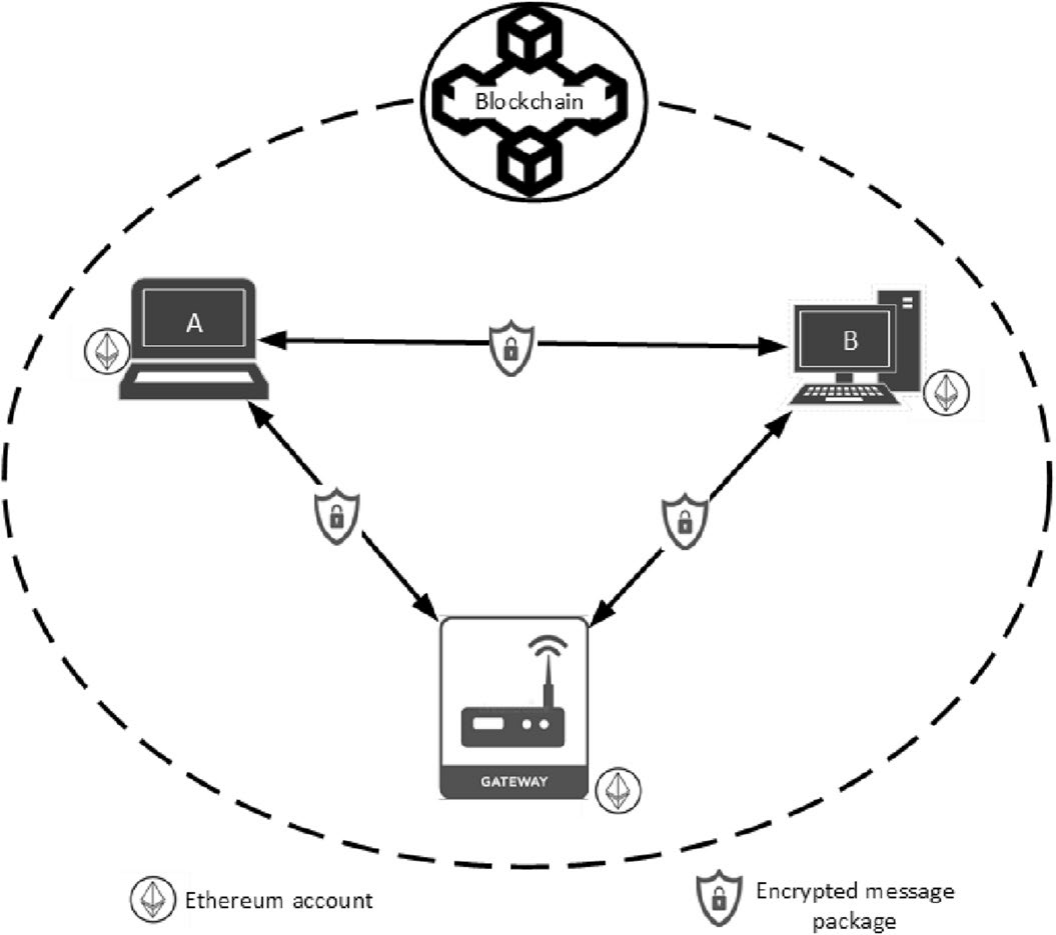
Proposed system model.

From Fig. 1, given a message package $x$ to be transmitted from a given node say $A$ to another say $B$ through a transparent private network of Ethereum blockchain, with $A$ and $B$ having Ethereum address of ${EA}_{A}$ and ${EA}_{B}$ respectively. We presumed that both $A$ and $B$ are registered and administered through an administrative node referred here as the gateway. However, the gateway has no significant influence during communications between $A$ and $B$. Thus, interaction between $A$ and $B$ is absolutely peer-to-peer and distributed.

According to Fig. 1, the role of the scheme is to encrypt the message package $x$, by considering the bit string representation of $x$ as an element of ${Z}_{n}=\{\mathrm{0,1},2,\dots ,n-1\}$ where $n$ is the number of elements in the set of ${Z}_{n}$. Consequently, the binary value of the data package $x$ must be less than $n$. The same holds for the ciphertext of the encrypted data package.

Given the set of public exponents $E=\{{e}{\prime}, {e}^{{\prime}{\prime}}, {e}^{{\prime}{\prime}{\prime}}\dots \varphi \left(n\right)\}$, set of private exponents $D=\{{d}{\prime}, {d}^{{\prime}{\prime}}, {d}^{{\prime}{\prime}{\prime}}\dots ,\varphi \left(n\right)\}$ and some large primes $p$ and $q \ni$ $n=p\cdot q$, then the generator function ($\varphi \left(n\right))$ of these primes can be computed as follows:

1 $$\varphi \left(n\right)=\left(p-1\right)\left(q-1\right),$$

where $$p\in {Z}_{p}$$, $$q\in {Z}_{q},$$ $${Z}_{q}\subseteq {Z}_{p}$$, and $${Z}_{p},{Z}_{q}\subseteq {Z}_{n} \ni$$ the order of both $${Z}_{p}$$ and $${Z}_{q}$$ has at least 1024 bits each. Similarly, each public exponent $${e}^{a}\in \left\{\mathrm{1,2},\dots ,\varphi \left(n\right)-1\right\}\ni$$ $$gcd\left({e}^{a},\varphi \left(n\right)\right)=1$$. This is to ensure that $$\exists {\left({e}^{a}\right)}^{-1} mod\, \varphi \left(n\right)$$, given rise to a private component $${d}^{a}$$. Thus, each private component ($${d}^{a})$$ can be computed as follows:

2 $${d}^{a}\cdot {e}^{a}=mod\, \varphi \left(n\right).$$

Assuming $${e}{^\prime}$$ and $${e}^{{\prime}{\prime}}$$ are both small prime values in $$E$$, then let node $$A$$ be a message sender that chooses a pair of integers $$n$$ and $${e}{\prime}$$ as its public key $$\ni$$ $${A}_{pub}=\left(n,{e}{\prime}\right);$$ and let $${d}{\prime}$$ be a private key of node $$A$$ $$\ni {A}_{prv}={d}{\prime}$$. Similarly, let $$B$$ be a message receiver that computes its public key, $${B}_{pub}=\left(n,{e}^{{\prime}{\prime}}\right),$$ as well as its private key, $${B}_{prv}={d}^{{\prime}{\prime}}$$. Given the Ethereum addresses ($$EAs)$$ $$\ni {{EA}_{A}}{\prime}\in A$$ and $${{EA}_{B}}{\prime}\in B$$, then $$A$$ and $$B$$ both submit their public keys together with their $$EAs$$ to the private network administrator (e.g., a smart gateway) to register in the network. As per the broadcasting rule of the blockchain network, copies of one another's public keys and $$EAs$$ are likewise given to each other.

To achieve non-repudiation during message transmission in the network, we employ the use of digital signature in the scheme. Given an element $$\alpha \ni$$ $$ord\left(\alpha \right)=q$$, and an integer $${d}{\prime}\ni 0<{d}{\prime}<q$$. If the public parameter $$\beta$$ can be computed as $$\beta ={\alpha }^{{d}{\prime}} mod\, p$$, then to compute the signature of the encrypted massage, $$A$$ will computes its signing public key ($${SK}_{pub})$$ parameter as $${SK}_{pub}=(p,q,\alpha ,\beta ,{EA}_{A})$$, while the signing private key $$({SK}_{prv})$$ parameter as $${SK}_{prv}=({d}{\prime})$$. Then, $$A$$ send the $${SK}_{pub}$$ to $$B$$ through broadcasting using $$B$$’s $$EA$$.

### Adversary model and assumptions

The following characteristics reflect the presumed capabilities of our adversaries in this study.

- It is presumed that an adversary may attempt to exploit the public exponents to determine or change the ciphertext, particularly when smaller ${e}^{a}$ values were used.
- An adversary can try to estimate the ephemeral key ${A}_{key}$ or calculate the signing private key ${SK}_{prv}$ by computing the large cyclic group discrete logarithm problem, or even by exploiting the subgroup as opposed to the whole cyclic group.
- An adversary can also attempt a man-in-the-middle or replay attacks by changing the Ethereum address or any of the signing public key parameters.
- It is presumed that the adversary cannot manipulate the system block creation process, which would compromise the blockchain.
- It is presumed that the network nodes are not resource constrained, thus, they can communicate in the large private network.

## The proposed model

**Figure 2:**
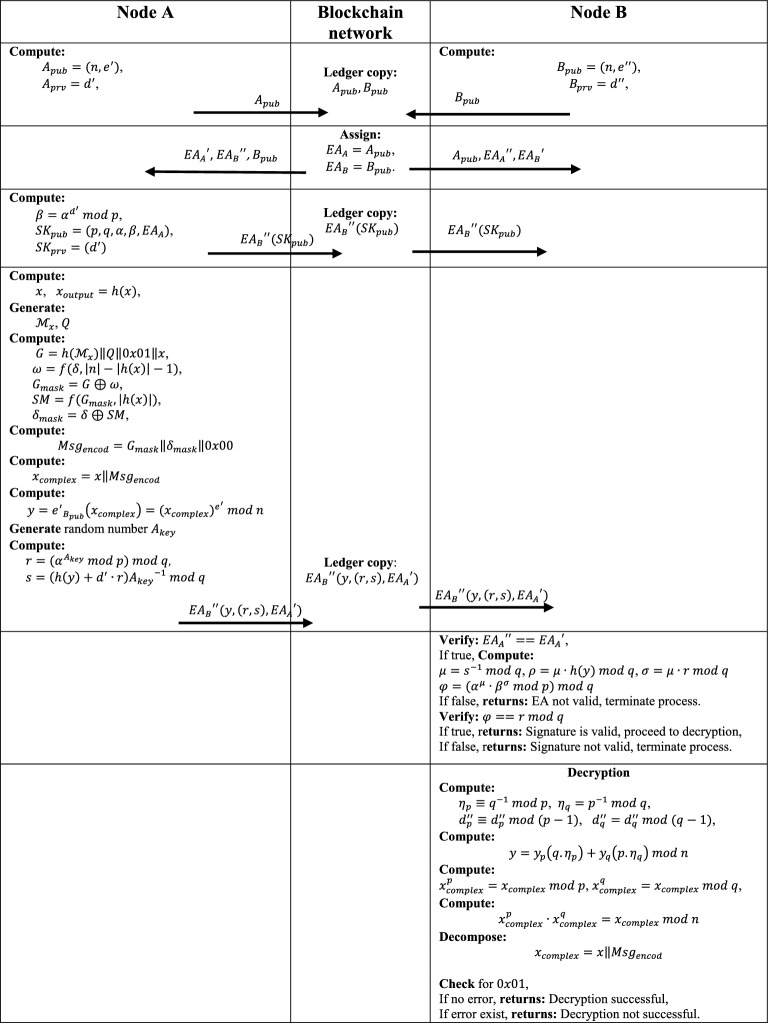
Details of the processes involved in the proposed model.

This section describes the structure and fundamental modeling of the proposed model. The section begins with Subsection “Message packaging and padding modeling”, which describes how the padding scheme was initialized to encapsulate the message and to help make the RSA cryptography scheme significantly more secure. Similarly, Subsections “Message encryption and signing modeling” and “Signature verification and decryption modeling” explain how the RSA cryptography scheme, digital signatures, and Ethereum addresses were utilized concurrently to ensure the privacy and security of the message package during transit. Figure 2 depicts a summary of the model's overarching sequential processes. In this research, it was assumed that the parameters $x, y, n, {d}{\prime}$ and ${d}^{{\prime}{\prime}}$ are very big values, often 1024 bits or more. The public exponents ${e}{\prime}$ and ${e}^{{\prime}{\prime}}$ are small prime numbers with a low hamming weight to facilitate a rapid encryption procedure inside the system.

### Message packaging and padding modeling

$A$ will first compute its message (message package) $x$, given the cardinalities of modulus $n=\left|n\right|$ and that of $x=\left|x\right|$ expressed in bytes, $A$ will then generate a hash of the message as ${x}_{output}=h(x)$ with cardinality $\left|h(x)\right|=160\mathrm{bits}$. Then, it generates string label ${\mathcal{M}}_{x}$ associated with $x$, and additional string $Q$ with length $\left|n\right|-\left|x\right|-2\left|h\left(x\right)\right|-2$. It, then, computes the parameter $G$ of length $\left|n\right|-\left|h\left(x\right)\right|-1$ in bytes, and another parameter $\omega$ as follows:

3 $$G=h\left({\mathcal{M}}_{x}\right)\Vert Q\Vert 0x01\Vert x,$$

4 $$\omega =f\left(\delta , \left|n\right|-\left|h\left(x\right)\right|-1\right),$$

where $0x01$ is single byte hexadecimal value, $\delta$ is a generated random seed value and $f(.)$ is the mask generation function. Given that ${G}_{mask}=G\oplus \omega$, $SM=f({G}_{mask},\left|h\left(x\right)\right|)$ and ${\delta }_{mask}=\delta \oplus SM$, then, $A$ will computes an encoded message string ${Msg}_{encod}$ of same cardinality with $\left|n\right|$ as follow:

5 $${Msg}_{encod}={G}_{mask}\Vert {\delta }_{mask}\Vert 0x00,$$

where $0x00$ is single byte hexadecimal value.

### Message encryption and signing modeling

To achieve privacy of the message package, $A$ computes message parameter ${x}_{complex}=x\Vert {Msg}_{encod}$ , and then, use the public key of $B$ to encrypt the message ${x}_{complex}$ to have a ciphertext $y$. Thus, $y$ can be generated as follow:

6 $$y={{e}{\prime}}_{{B}_{pub}}\left({x}_{complex}\right)={\left({x}_{complex}\right)}^{{e}{\prime}} mod \,n,$$

$$where x,y\in {Z}_{n}$$

It is essential to notice that the system is still safe even though the prime public exponent is so small, since the private exponent remains sizable.

To protect the ciphertext from active attacks such as man-in-the-middle (MITM), impersonation, replay, and session hijacking, a signature must be added at this point. To accomplish this, a digital signature scheme and a unique Ethereum address (EA) of 20 bytes were employed. Since all nodes are presumed to have previously registered and documented their Ethereum addresses in the network, all internal message communication within the model will be signed securely on the blockchain network using their EAs, protecting it against MITM and replay threats.

Assuming that the signature of the encrypted massage $y$ consists of a pair of integers such as $\left(r,s\right),$ each having a length of 160 bit, making a total of 320-bit length; thus, if an ephemeral integer key ${A}_{key}$ is chosen at random such that $0<{A}_{key}<q$, then $r,s$ can be computed as follow:

7 $$r=\left({\alpha }^{{A}_{key}} mod \,p\right)mod \,q$$

8 $$s=\left(h\left(y\right)+{d}{^\prime}\cdot r\right){{A}_{key}}^{-1} mod\, q.$$

From Eq. (8), a 160-bit signature can be obtained by hashing the encrypted message $y$ using SHA-1 hash function. Such signature can also be described as the representative of the message $x{, Msg}_{encod}$. With this, encrypted message $y$, the signature $\left(r,s\right)$ and ${{EA}_{A}}{\prime}$ of $A$ are then sent to the receiver $B$ as an encrypted message string using $B$’s $EA$: ${{EA}_{B}}^{{\prime}{\prime}}(y,\left(r,s\right),{{EA}_{A}}{\prime})$.

### Signature verification and decryption modeling

On receipt of the encrypted message string, $B$, decrypt it and verifies the signature as follows:

Initially, $B$ checks if ${{EA}_{A}}^{{\prime}{\prime}}=={{EA}_{A}}{\prime}$, if true, then it computes some auxiliary parameters $\mu ,\rho ,$ and $\sigma$ as: $\mu ={s}^{-1} mod\, q$, $\rho =\mu \cdot h(y) mod \,q$ and $\sigma =\mu \cdot r \,mod \,q$. With this, $B$ then computes another auxiliary parameter $\varphi$ as:

9 $$\varphi =\left({\alpha }^{\rho }\cdot {\beta }^{\sigma }\, mod\, p\right)mod \,q .$$

Let ${ver}_{{SK}_{pub}}(y,\left(r,s\right))$ be a verification function that checks whether $\varphi =r\, mod \,q$ by $B$. As a result, the signature $(r,s)$ will be accepted only if the above expression is true; otherwise, the signature is invalid.

$$\varphi \left\{\begin{array}{c}=r \,mod\, q \Rightarrow the\, signature\, is\, valid \\ \ne r \,mod \,q \Rightarrow the \,siganture\, is\, not \,valid\end{array}\right.$$

If the signature is returned to be valid, then decryption is performed by conducting an inverse transformation on the encrypted message and exponentiation parameters, followed by an arithmetic transformation into the original message. Given the encrypted message $y$ and the prime integers $p$ and $q$, then from the principle of Chines Remainder Theorem (CRT)^44,45^, given the coefficients ${\eta }_{p}$ and ${\eta }_{q}$ defined as ${\eta }_{p}={q}^{-1} mod\, p$, and ${\eta }_{q}={p}^{-1} mod\, q$ respectively, the inverse transformation of $y$ can be represented as follows:

10 $$y={y}_{p}\left(q.{\eta }_{p}\right)+{y}_{q}\left(p.{\eta }_{q}\right) mod\, n,$$

where ${y}_{p}$ and ${y}_{q}$ are modular form of $y$ and they are given as: ${y}_{p}={x}_{p}^{{d}_{p}^{{\prime}{\prime}}} mod \,p$ and ${y}_{q}={x}_{q}^{{d}_{q}^{{\prime}{\prime}}} mod \,q$. Where ${d}_{p}^{{\prime}{\prime}}$ and ${d}_{q}^{{\prime}{\prime}}$ are the decryption exponent bounded by the two prime integers p and q, and they are given as: ${d}_{p}^{{\prime}{\prime}}={d}_{p}^{{\prime}{\prime}} mod \,(p-1)$ and ${d}_{q}^{{\prime}{\prime}}={d}_{q}^{{\prime}{\prime}} mod \,(q-1)$. Thus, the modular form of ${x}_{complex}$ can be generated as: ${x}_{complex}^{p}={x}_{complex} mod\, p$ and ${x}_{complex}^{q}\equiv {x}_{complex} mod \,q$.

Since $p,q\in {Z}_{n}$, then by combining ${x}_{complex}^{p}$ and ${x}_{complex}^{q}$, we have:

11 $${x}_{complex}^{p}\cdot {x}_{complex}^{q}={x}_{complex} mod\, n.$$

Then, the parameter ${x}_{complex}$ will be decomposed to give rise to $x$ and ${Msg}_{encod}$. The recipient will now examine the structure of the decoded message. A decryption error will occur when a byte of $0x01$ hexadecimal value does not exist to distinct $Q$ and $x$. Returning a decryption failure to the recipient (or a possible adversary) should never divulge anything about the plaintext. Furthermore, suppose $n$ contains $t+1$ bits, the length of $p$ and $q$ is about $t/2$ bits, where $t$ is the modulus $n$ bit length. The bounds of $p$ and $q$ are applicable to the sizes of all integers employed in the exponentiations. Using the square-and-multiply method, each operation requires around $1.5t/2$ modular arithmetic operations, making it four times faster than a $t$-bit operations^12^. Figure 2 provides the details of the processes involved in the proposed system.

## Security analysis

This section analyzes how the proposed model addresses fundamental security and privacy issues considering the proposed adversary model to establish how effectively the proposed model is protected.

### Theorem 1

The modest public exponents used and the ciphertext created from the message package are neither deterministic nor changeable. Therefore, the adversary cannot estimate the public exponent or change the ciphertext into another ciphertext that results in a known modification of the plaintext.

### Proof

The model described utilizes the Optimal Asymmetric Encryption Padding (OAEP) approach. To prevent change of the ciphertext or simple guessing of the public exponent, the approach embeds a random structure before encrypting the data. During decryption, the recipient of the message will always examine its structure. If a byte of $0x01$ hexadecimal value does not exist to distinct $Q$ and $x$, a decryption error will occur. The return of a decryption failure to the receiver (or a potential adversary) should never disclose the plaintext. Similarly, the proposed model is safe even with such small public exponents since the private exponent still has the entire bit length $t+1$ in general.

### Theorem 2

The proposed model is secure against an adversary attempting to estimate the ephemeral key ${A}_{key}$ or calculate the signing private key ${SK}_{prv}$ by computing the large cyclic group discrete logarithm problem, or even attempting to exploit the subgroup as opposed to the whole cyclic group.

### Proof

To avoid ephemeral key estimation, the proposed architecture ensures to generate and use a new random key ${A}_{key}$ in each signature operation. In addition, the model employs a $p$ of at least 1024 bits in length. It is estimated that this level of security provides 80 bits, therefore an attack would need around ${2}^{80}$ operations. Even if the adversary attacks the subgroup of order $q$ rather than the whole cyclic group, they cannot possess sufficient computational resources to exploit the subgroup feature. This is because the subgroup in issue has an estimated order of ${2}^{160}$, resulting in a level of security equal to $\sqrt{{2}^{160}}$. Since the size of the subgroup never decreases, effective exploitation is made more difficult, resulting in a complexity of ${2}^{80}$. Moreover, because the number of bits in the hash output defines the security level of a hash function, it is difficult for an adversary to solve the discrete logarithm problem to match the security level of the hash function.

### Theorem 3

During message transmission, the proposed model assures transactional data privacy, secure and genuine provenance. Therefore, an adversary cannot affect the transmission channel or the message on transit.

### Proof

In the proposed architecture, a unique 20-byte Ethereum address is used, and it is given instantaneously to all network nodes with no collision at the time the node joins the network. Consequently, all nodes are presumed to have been previously registered and documented in the network using their individual Ethereum addresses. This sophisticated blockchain feature is used in conjunction with the previously established public key infrastructure mechanism are used to achieve transactional data surety and privacy in the network. Each EA in Ethereum has its own set of asymmetric keys, and the network can be configured to use secured socket layer (SSL) for all node-to-node connections, ensuring perfect privacy. Furthermore, to deceive other network nodes, the adversary may potentially impersonate a legitimate node and transmit them false data. However, every piece of internal message communication inside the model is signed securely on the blockchain network, safeguarding it against MITM and replay threats. Furthermore, the use of public key, signing public key parameters, and EA in the verifications prevents MITM and replay attacks. As the adversary's EA varies from the actual EA used in conjunction with the initial public key and signing public key parameters, his signature is invalid. In addition to being safe against MITM and replays attacks, the created events are also tamper-proof and validated by smart contracts.

## Performance analysis

This section compares the performance of the proposed model to that of competing and relevant previously published approaches in Refs.^4,32^. In an Ethereum blockchain network, the proposed model makes considerable use of digital certificates and accelerated PKI. The section presents a comparative analysis of execution time, communication cost, and storage cost before concluding with a comparative analysis of the security characteristics relevant to this research.

### Execution time

According to Refs.^46–48^, the estimated execution time in milliseconds of the cryptographic procedure was determined using an Intel^®^ Core TM i5-7200 CPU @ 2.7 GHz, 16.0 GB RAM, and Windows 10 64-bit operating system, together with the Visual Studio 2008 programme and the MIRACL C/C +  + library. Additionally, methods like advanced-encryption standard (AES) (128 bit), RSA (1024 bit), secure-hash algorithm 1 (SHA-1) (160 bit) and elliptic-curve (EC) cryptosystem (320 bit) were used to test assumed period.

Recall that the proposed model employs an enhanced speed-up approach that accelerates the encryption process by a factor of approximately 17 since a modest and safer value of ${e}{\prime}$, ${2}^{16}+1$, was considered. In addition, the decryption process is accelerated by a factor of 4 since the complexity of multiplication falls quadratically with the bit length. Thus, the average overall acceleration achieved was factor 16.5. Hence, the execution time for a modular-exponential computation (${T}_{me}$) in our model is 0.0969 ms ($ms$), while it is 1.6003 ms for traditional processes without acceleration. Moreover, ${T}_{hash}$, ${T}_{mul}$, and ${T}_{ed}$ are hash function (0.0004 ms), point multiplication operations on elliptic curve (1.8269 ms) and symmetric key encryption/decryption (0.1303 ms), respectively.

From the results in Table 2, the proposed protocol requires a minimum execution time of $2.8822 ms$, as compared to the $4.9356$ and $14.2324 ms$ required by both benchmark models, respectively. This indicates that the proposed protocol is more secure and can run faster than the benchmark model.

**Table 2:** Execution time results.

	Total operation	Execution time ($ms$)
S-USI scheme^32^	$11{T}_{hash}+3{T}_{me}+1{T}_{ed}$	4.9356
PES scheme^4^	$6{T}_{hash}+4{T}_{me}+4{T}_{ed}+4{T}_{mul}$	14.2324
Proposed model	$23{T}_{me}+5{T}_{hash}+5{T}_{ed}$	2.8822

### Communication cost

According to Refs.^49–51^, the Ethereum address, RSA, ECC point, symmetric key encryption/decryption, hash function, random number, and identity were specified as 160, 1024, 320, 256, 160, 160, and 128 bits respectively. Thus, the proposed model’s message packaging and padding, message encryption and signing and signature verification and decryption phases need $\{160+160\}$ bits for the two exchanged messages $\{{{EA}_{B}}^{{\prime}{\prime}}\left({SK}_{pub}\right)\}$, and $\{{{EA}_{B}}^{{\prime}{\prime}}\left(y,\left(r,s\right),{{EA}_{A}}{\prime}\right)\}$. Thus, the protocol's total communication cost is 320 bits as depicted in Table 4. In contrast, the benchmarked S-USI method used three message rounds for transmission between User and a remote server: ECC points (P1, P2, P3), ECC points (P3, P4, P5) and hash data (H1, H2). Thus, the S-USI scheme^32^ overall communication cost was calculated: $\{960 + 960 + 320= 2240\}$ bits. Given that an Index (${I}_{t}$) value equals to 32 bits, in PES scheme^4^ two messages M1 and M2 were transmitted as {hash function, random number} and {hash function, Index (${I}_{t}$)}: {160 + 128 + 160 + 32 = 480} bits. The summary of the computational cost in all models is given in Table 3.

**Table 3:** Communication cost results.

	Total operation	Communication cost (bits)
S-USI scheme^32^	$\{960 + 960 + 320\}$	2240
PES scheme^4^	$\{160+128+160+32\}$	480
Proposed model	$\{160+160\}$	320

### Storage cost

To determine the storage cost associated with the proposed model during communication, the storage parameters ${{EA}_{B}}^{{\prime}{\prime}}({SK}_{pub})$ and ${{EA}_{B}}^{{\prime}{\prime}}(y,\left(r,s\right),{{EA}_{A}}{\prime})$ were considered, which have a total cost of $\{160 + 160 = 320 bits\}$ when added together. However, as shown in Table 4, the current schemes (the S-USI scheme^32^) has a storage cost of $\{160 + 160 + 256 + 160 + 160 = 896 bits\}$ and $\{160+160+160=480 bits\}$ respectively which are higher than that of the proposed model as shown in Table 4.

**Table 4:** Storage cost results.

	Total operation	Storage cost (bits)
S-USI scheme^32^	$\{160 + 160 + 256 + 160 + 160 \}$	$896$
PES scheme^4^	$\{160 + 160 + 160 \}$	$480$
Proposed model	$\{160 + 160 \}$	$320$

In a nutshell, the proposed protocol uses less computational power of $2.8822ms$, requires less communication overhead of $320 bits$ and less memory consumption of $320bits$ as compared with the existing models in Refs.^4,32^. This compensates for the IoT nodes' limited CPU processing capabilities and memory capacity.

### Comparative of security features

The proposed model and reference models were evaluated based on several security characteristics. From Table 5, neither of the benchmark models offered superior resistance to impersonation threats on nodes and known session-Secret temporary information attacks, nor could they guarantee transactional data privacy during communications or perfect forward secrecy of data in transit. However, the proposed model satisfies all security requirements when compared to reference models.

**Table 5:** Comparative of security features.

Security features	S-USI protocol^32^	PES scheme^4^	Proposed protocol
Resilience to impersonation threats on node	NO	NO	YES
Resilience to MITM and replay threats	YES	YES	YES
Resilience to privileged-insider threat	YES	YES	YES
Transactional data privacy	NO	NO	YES
Resilience to known session-Secret temporary information attack	NO	NO	YES
Resilience to anonymity and untraceability threats	YES	YES	YES
Perfect forward secrecy of data in transit	NO	NO	YES
Authentication	YES	YES	YES

## Discussions and future improvements

The proposed framework was developed using security features i.e., RSA cryptosystem, digital certificates, and private Ethereum blockchain to meet the security requirements of large private networks. Similarly, we present theoretical security and performance analyses to evaluate the viability of incorporating such security features into the proposed model. The proposed model is adaptable to the evolving needs of multiple smart city-based enterprises. Due to the confidence instilled by the encryption of data and transactions, users of large private networks are more likely to continue utilizing such a private blockchain-based system.

This study has three significant limitations. One is that the proposed method was only theoretically tested and compared to state-of-the-art models using theoretical computations and evaluations. The second concern is the blockchain's actual structure, such as its incapacity to scale, and the third is the behavior of stakeholders in the large private networks. Malicious activity in the context of large private networks is complicated and influenced by multiple factors; therefore, we plan to evaluate the proposed model with other relevant metrics, such as computational complexity, scalability, and robustness against various types of attacks, in a future extension that will include the model's full practical implementation.

Similarly, a more in-depth analysis of stakeholder behavior associated with large private networks will be conducted for the future extension. In addition, the influence of the proposed model on individual behavior in large private network settings cannot be demonstrated unless the model's essential properties and building elements are technologically realizable. Given that the fundamental issue with blockchain technology is its incapacity to scale, it is reasonable to presume that the solutions being developed to enhance blockchain technology's scalability will also be applicable to vast private networks. Scalability should therefore be one of the primary focuses of future development.

## Conclusion

This study introduces a privacy-preserving framework based on digital certificates, RSA-based PKI, and the Ethereum blockchain to address user transactional data privacy concerns and to guarantee that data in transit remains secure and unaltered and that its provenance remains authentic and secure during node-to-node interactions within a large private network. The proposed model has produced an increased speed up method that speeds the encryption process by about 17 times, while the decryption process is expedited by four times. Therefore, the average overall acceleration obtained was 16.5. We proved that the proposed framework is capable of theoretically preventing several vulnerabilities in large private networks, and that its performance is superior to that of prior approaches. The results of both the security and performance analyses indicate that the proposed framework can protect transactional data during communications on large private networks more effectively and securely than existing methods. Future expansion will entail evaluating the framework's scalability and usefulness by applying it to several large private network implementations in both simulation and real world.

## Data Availability

All data generated or analyzed during this study are included in this published article.
